# Crohn’s and Colitis Canada’s 2021 Impact of COVID-19 and Inflammatory Bowel Disease in Canada: Seniors With IBD

**DOI:** 10.1093/jcag/gwab025

**Published:** 2021-11-05

**Authors:** Charles N Bernstein, Harminder Singh, Sanjay K Murthy, Geoffrey C Nguyen, Eric I Benchimol, Alain Bitton, M Ellen Kuenzig, James Guoxian Huang, Jennifer L Jones, Kate Lee, Laura E Targownik, Joseph W Windsor, Mariam S Mukhtar, Parul Tandon, Gilaad G Kaplan

**Affiliations:** 1 Department of Internal Medicine, Max Rady College of Medicine, Rady Faculty of Health Sciences, University of Manitoba, Winnipeg, Manitoba, Canada; 2 IBD Clinical and Research Centre, University of Manitoba, Winnipeg, Manitoba, Canada; 3 Department of Medicine, The Ottawa Hospital IBD Centre, University of Ottawa, Ottawa, Ontario, Canada; 4 Mount Sinai Hospital Inflammatory Bowel Disease Centre, University of Toronto, Toronto, Ontario, Canada; 5 SickKids Inflammatory Bowel Disease Centre, Division of Gastroenterology, Hepatology and Nutrition, The Hospital for Sick Children, Toronto, Ontario, Canada; 6 Child Health Evaluative Sciences, SickKids Research Institute, Toronto, Ontario, Canada; 7 ICES, Toronto, Ontario, Canada; 8 Department of Paediatrics and Institute of Health Policy, Management and Evaluation, University of Toronto, Toronto, Ontario, Canada; 9 Department of Medicine, McGill University Health Centre, McGill University, Montreal, Quebec, Canada; 10 Department of Medicine, Dalhousie University, Halifax, Nova Scotia, Canada; 11 Crohn’s and Colitis Canada, Toronto, Ontario, Canada; 12 Division of Gastroenterology and Hepatology, Mount Sinai Hospital, University of Toronto, Toronto, Ontario, Canada; 13 Department of Medicine, University of Calgary, Calgary, Alberta, Canada; 14 Department of Community Health Sciences, University of Calgary, Calgary, Alberta, Canada; 15 Department of Internal Medicine, King Abdulaziz University Hospital, Jeddah, Saudi Arabia

**Keywords:** Coronavirus, Crohn’s disease, Elderly, SARS-CoV-2, Senescence, Ulcerative colitis

## Abstract

The risk of hospitalization and death from Coronavirus disease-19 (COVID-19) increases with age. The extreme elderly have been particularly vulnerable, with those above the age of 80 having a case-fatality rate as high as 15%. Aging of the immune system can lead to impaired inflammatory responses where eradication of an organism such as Severe Acute Respiratory Syndrome CoronaVirus 2 (SARS-CoV2) is inadequate but is exaggerated in such a way as to enhance pneumonia and acute respiratory distress syndrome. Frailty and comorbidity are both more common in the elderly, and these can enhance the morbidity and mortality from COVID-19. Studies from Northern California and Italy suggest that elderly persons with inflammatory bowel disease (IBD) were more likely to acquire SARS-CoV-2 infection than youths with IBD. While the specific impact of age-related comorbidity is less well established among people with IBD who acquire COVID-19, data from the Surveillance Epidemiology of Coronavirus Under Research Exclusion (SECURE-IBD) database reported that having two or more chronic illnesses was independently associated with developing severe COVID-19 among people with IBD. Despite having exaggerated auto-inflammatory responses, people with IBD do not appear to have an overall increased risk of developing severe COVID-19 than the general population. However, whether seniors with IBD do worse once they acquire COVID-19 compared with seniors without IBD is not known. The advent of telehealth care has posed an information technology challenge for many seniors with and without IBD. Most persons with IBD have expressed satisfaction with virtual IBD health care (phone or video-based visits). While the elderly may have less robust immune responses to vaccinations, learning from experiences with other vaccination programs, especially influenza, have shown that vaccinating seniors decreases both morbidity and mortality and, in turn, healthcare resources.

**Key Points**

- Seniors have the highest risk of severe COVID-19 in the general population and among those with IBD.
- Senescence of the immune system and comorbidities both enhance the morbidity and mortality from COVID-19 among the elderly.
- Seniors with IBD do not have an increased risk of acquiring SARS-CoV-2 and share a similar risk of complications from COVID-19 as compared to seniors without IBD.
- Telehealth care has been an important mechanism for seniors with and without IBD to maintain contact with their healthcare providers and ensure their care unrelated to COVID-19 is minimally compromised by the pandemic.
- While seniors may have less robust immune responses to vaccinations, experiences with other vaccination programs, especially influenza, have shown that vaccinating seniors decreases both morbidity and mortality and, in turn, healthcare resources.

## INTRODUCTION

Seniors represent the Fastest-growing demographic with IBD in Canada with nearly 1 in 160 Canadians above the age of 65 affected (1,2). Every day, new diagnoses of IBD are being made in seniors, and the prevalent IBD population is aging. Consequently, gastroenterology clinics today are contending with an older population and having to balance care of IBD with age-related comorbidities, such as diabetes, cardiovascular disease (CVD) and dementia (3). Understandably, the Coronavirus disease-19 (COVID-19) pandemic created significant anxiety among seniors with IBD as the morbidity and mortality of COVID-19 disproportionately impacted those of advanced age. This section reviews the impact of COVID-19 on people with IBD living in Canada.

## COVID-19 AND THE ELDERLY

The risk of hospitalization and death from COVID-19 increases with age (4–6). In the first half of the COVID-19 pandemic in the United States, people above 65 years of age comprised 45% of hospitalizations, 53% of intensive care unit admissions and 80% of deaths associated with COVID-19, despite comprising just 17% of the population (7). The extreme elderly have been particularly vulnerable, with those above the age of 80 having a case-fatality rate as high as 15% (8). Immune aging, frailty, and comorbid illnesses may all contribute toward an increased risk of adverse COVID-19-related outcomes among the elderly.

Immune aging (immunosenescence) refers to age-related changes that tip the balance of immunity in favour of pro-inflammatory pathways. The expression of angiotensin-converting enzyme 2 (ACE2), which converts angiotensin II to angiotensin, decreases with age and with CVD (9–11). Angiotensin II has pro-inflammatory properties that may mediate acute lung injury through vasoconstriction and increased vascular permeability. ACE2 is expressed in the lung, heart, kidney, blood vessels, brain, intestine and fat tissue. As ACE2 is also the receptor for the severe acute respiratory syndrome coronavirus 2 (SARS-CoV-2) spike protein (12), reduced ACE2 expression in these organs could lead to exaggerated inflammatory responses in the elderly in response to SARS-CoV-2 infection, especially in those with CVD.

Aging is also characterized by chronic, low-grade inflammation with a relative increase in the production of pro-inflammatory cytokines, such as interleukin (IL)-6 and tumor necrosis factor-α (TNF-α) (13,14). Severe cases of COVID-19, including people requiring intensive care admission, showed higher levels of TNF-α, among other proinflammatory cytokines like IL-6 (15,16). Aging may further be associated with reduced levels of type I interferons (i.e., interferons α and β); these are important for lowering the susceptibility of cells neighboring virus-infected cells from viral entry and replication as well as for activating natural killer cells and Th1-lymphocytes, which amplify the anti-viral response (17–19). Together, these pro-inflammatory changes might contribute to the development of acute respiratory distress syndrome and multi-organ failure in the elderly (20).

Frailty, as reflected by reduced physiologic reserve due to comorbidities, bone and muscle atrophy, decreased physical activity and reduced cognition, is more prominent in seniors. Greater frailty is associated with increased mortality and morbidity, particularly in the setting of systemic infection (21–24). Frailty has also been correlated with higher levels of IL-6, TNF-α and C-reactive protein, suggesting an association with a pro-inflammatory state (25,26). These frailty factors may place elderly persons at further risk of severe outcomes with COVID-19 infection.

Pre-existing CVD, chronic lung disease, hypertension, diabetes and obesity, which are observed more often in seniors, have all been associated with more severe COVID-19 (8,27–29). While the relationships between these conditions and severe COVID-19 are not well understood, it stands to reason that they may predispose to impaired adaptive immunity, increase in pro-inflammatory cytokines and poorer vascular and respiratory reserve to combat infection, leading to greater viral replication, maladaptive immune responses, overwhelming inflammation and multi-organ failure. Furthermore, in the context of greater atherosclerotic disease, severe COVID-19 infection may increase the risk of cardiac and neurologic complications, such as stroke and myocardial infarction (30–34). Additionally, COVID-19 is associated with an increase in arterial and venous thrombotic complications, which may be even more pronounced in the elderly due to underlying atherosclerotic disease (35–37).

## COVID-19 AND THE ELDERLY WITH IBD

A growing proportion of people with IBD are elderly, and this already vulnerable group may shoulder the brunt of the risk from COVID-19. Despite physical isolation, seniors with IBD seemed to have managed relatively well with respect to mental health compared with younger persons with IBD. The brave new realm of virtual medicine that evolved during the COVID-19 pandemic has also introduced new challenges for the elderly as well as opportunities.

As in the general population, the risk of severe COVID-19 and disease-related mortality is increased in seniors with IBD (38). Certain chronic diseases also increase the risk of COVID-19 severity. Reassuringly, a Swedish population-based study showed that IBD diagnosis among those aged ≥60 years neither increased the risk of hospitalization with COVID-19 nor COVID-19 severity compared with the general population (39). While the specific impact of age-related comorbidity is less well established among people with IBD who acquire COVID-19, data from the Surveillance Epidemiology of Coronavirus Under Research Exclusion (SECURE-IBD) registry suggest that having two or more chronic illnesses was independently associated with developing severe COVID-19 among people with IBD (e.g., CVD, diabetes, lung disease, hypertension, cancer, history of stroke, chronic kidney disease, chronic liver disease and others) (38). Studies from Italy and the Netherlands have similarly shown that comorbidity burden is an independent predictor of adverse COVID-19 outcomes among people with IBD (40,41). Notably, despite having exaggerated auto-inflammatory responses, persons with IBD do not appear to have an overall increased risk of developing severe COVID-19 than the general population (38,42–44). However, a study from Northern California did suggest that persons with IBD older than 66 years were more likely to acquire SARS-CoV-2 infection than younger persons with IBD (45). Another Italian study also showed that elderly persons with IBD (≥65 years) had a nearly sixfold higher risk of acquiring COVID-19-related pneumonia compared with non-elderly persons with IBD (40). Seniors with IBD, in general, are less likely to be on immunosuppressants, though corticosteroid use is similar (46). Data from the SECURE-IBD registry suggest that while the use of corticosteroids and thiopurines did increase the risk of severe COVID-19, the use of biologics, especially anti-TNF agents, may be protective (47). Overall, IBD diagnosis does not appear to increase the risk of SARS-CoV-2 infection or severe COVID-19 in the elderly population by itself.

The COVID-19 pandemic has also had a substantial impact on the mental health of people with IBD in general, with the majority citing negative impact on mood, anxiety and sleep. Interestingly, in a survey from the United Kingdom, individuals with IBD aged above 55 years were more likely to experience a positive psychosocial impact (48). In another Australian survey of people with IBD, younger age was actually associated with a greater prevalence of depression and anxiety (49). Thus, despite social isolation, elderly persons with IBD seemed to have coped better relative to their younger counterparts.

Beyond the immediate risk posed by COVID-19 to seniors with IBD, another challenge has been accessing continued medical care during lockdowns and business occupancy limits. Throughout the course of the COVID-19 pandemic, IBD health services have transitioned to virtual care throughout most of Canada. Most of the persons with IBD have expressed satisfaction with virtual IBD health care (phone or video-based visits) (50,51). For older persons with IBD, adapting to new technology may have posed a substantial challenge. However, the flexibility to utilize phone visits and a variety of public, video-based platforms (e.g., Facetime, Skype and Zoom) has made this transition easier. Many individuals with IBD and physicians have expressed a desire to continue telemedicine beyond the pandemic, and this may benefit the elderly with IBD who may have baseline limited mobility.

## VACCINES AND THE ELDERLY

Similar to COVID-19, the vast majority of seasonal influenza infections and related deaths from the disease occur among the elderly. A Dutch study between 1967 and 1989 reported that 95% of influenza-related mortality occurred among people above 60 years of age (52). Hence, seniors are a critical group to vaccinate against COVID-19. However, with senescence of the immune system, especially after the age of 60, there is the potential for reduced response to vaccines (53). What to expect in the elderly population from COVID-19 vaccines can be drawn from previous studies on influenza vaccine experiences.

Some studies have suggested that influenza vaccination may be less effective among subgroups of senior populations. For example, a multicentre study suggested that influenza vaccine effectiveness is lessened among frail older adults (54). Influenza outbreaks have been reported in nursing homes with excellent vaccine coverage (55). However, even with a lower vaccine efficacy, influenza vaccines prevent a large number of hospitalizations, intensive care unit admissions and deaths in the older adult population. For example, in a modelling study, during the 2012–2013 influenza season, a vaccine with 10% effectiveness and 66% coverage was estimated to avert approximately 13,000 hospitalizations among individuals ≥65 years of age in the United States; a vaccine with 40% effectiveness and the same coverage was estimated to avert 60,000 hospitalizations (56). A systematic review for the Cochrane collaboration reported that older adults receiving the influenza vaccine experience less influenza over a single season (2.4% from 6% [Risk Ratio: 0.42; 95% confidence interval: 0.27,0.66]) (57).

On the one hand, similar results of lower efficacy among individuals above 80 years have been reported for pneumococcal vaccination (58). On the other hand, the herpes zoster vaccine efficacy is minimally affected by age (59). Additionally, individuals with IBD—particularly those treated with combination thiopurines and tumor necrosis antagonist therapy—have impaired immunologic response to vaccines, including against influenza (60). There are, however, no differences in adverse effects among vaccinated individuals with or without IBD (61). The CLARITY-IBD study evaluated a subpopulation of individuals with IBD who were above the age of 60 and vaccinated with messenger ribonucleic acid (mRNA) or non-viral vector vaccine. Age above 60 years was an independent predictor of lower anti-SARS-CoV-2 antibodies (62).

Extrapolating from these findings, the effectiveness of COVID-19 vaccination among the elderly with IBD may be lower than among younger individuals, particularly compared with those without IBD. However, substantial benefits are likely to occur. A necessary strategy to improve benefits among elderly individuals with IBD is going to be avoiding delays in receiving the second dosage.

## Conclusions

The COVID-19 pandemic has disproportionately impacted the senior population. Those with IBD who are elderly may be at increased risk of acquiring SARS-CoV-2 infection and, like the general population, have similarly higher risk of more severe disease and mortality. Despite these risks, seniors with IBD seemed to have fared no worse—if not better—than their younger IBD counterparts from a mental health perspective. Most have adapted reasonably well to virtual care, and there is an opportunity to continue some aspects of these transformative models of healthcare delivery to address the specific needs of this population post pandemic.
